# Biobanking in health care: evolution and future directions

**DOI:** 10.1186/s12967-019-1922-3

**Published:** 2019-05-22

**Authors:** Luigi Coppola, Alessandra Cianflone, Anna Maria Grimaldi, Mariarosaria Incoronato, Paolo Bevilacqua, Francesco Messina, Simona Baselice, Andrea Soricelli, Peppino Mirabelli, Marco Salvatore

**Affiliations:** 1 IRCCS SDN, Naples Via Emanuele Gianturco, 11, 80143 Naples, Italy; 2 Ospedale Evangelico Betania, Naples, Italy; 3 Department of Sport Sciences & Healthiness, University of Naples Parthenope, Naples, Italy

**Keywords:** Biobank, Biobanking, Imaging biobank, Personalized medicine, Human samples, Bioethics, Radiomics, Radiogenomics

## Abstract

**Background:**

The aim of the present review is to discuss how the promising field of biobanking can support health care research strategies. As the concept has evolved over time, biobanks have grown from simple biological sample repositories to complex and dynamic units belonging to large infrastructure networks, such as the Pan-European Biobanking and Biomolecular Resources Research Infrastructure (BBMRI). Biobanks were established to support scientific knowledge. Different professional figures with varied expertise collaborate to obtain and collect biological and clinical data from human subjects. At same time biobanks preserve the human and legal rights of each person that offers biomaterial for research.

**Methods:**

A literature review was conducted in April 2019 from the online database PubMed, accessed through the Bibliosan platform. Four primary topics related to biobanking will be discussed: (i) evolution, (ii) bioethical issues, (iii) organization, and (iv) imaging.

**Results:**

Most biobanks were founded as local units to support specific research projects, so they evolved in a decentralized manner. The consequence is an urgent needing for procedure harmonization regarding sample collection, processing, and storage. Considering the involvement of biomaterials obtained from human beings, different ethical issues such as the informed consent model, sample ownership, veto rights, and biobank sustainability are debated. In the face of these methodological and ethical challenges, international organizations such as BBMRI play a key role in supporting biobanking activities. Finally, a unique development is the creation of imaging biobanks that support the translation of imaging biomarkers (identified using a radiomic approach) into clinical practice by ensuring standardization of data acquisition and analysis, accredited technical validation, and transparent sharing of biological and clinical data.

**Conclusion:**

Modern biobanks permit large-scale analysis for individuation of specific diseases biomarkers starting from biological or digital material (i.e., bioimages) with well-annotated clinical and biological data. These features are essential for improving personalized medical approaches, where effective biomarker identification is a critical step for disease diagnosis and prognosis.

## Background

In a 1996 paper investigating the role of oxidative DNA damage as an independent risk factor in cancer, Loft and Poulsen first used the word “biobank” to refer to the use of human biological material [1]. Since then, the biobanking field has grown and improved the conduct of medical research. Much of this progress occurred following the advent of -omics science (genomics, transcriptomics, proteomics, metabolomics) and the ability to develop large electronic databases that store huge amounts of information (big data) associated with patient clinics [2]. In this way, biobanks have a primary role in the era of precision medicine, which is based on analyzing samples with clinical data. The availability of a large collection of patient samples (with well-annotated patient clinical and pathological data) is a critical requirement for personalized medicine. If more high-quality samples are available through biobanks, researchers will be able to use these resources to advance patient treatment [3].

In this context, the Organization for Economic Cooperation and Development defined biobanks as structured resources that can be used for the purpose of genetic research, including human biological materials and/or information generated from genetic analysis and associated information [4]. The European Commission published a comprehensive document highlighting the primary roles of a biobank: (i) to collect and store biological materials annotated with medical data and often epidemiological data; (ii) not consider collection projects static but continuous or long term; (iii) to associate with current and/or future research projects at the time of specimen collection; (iv) to apply coding or anonymization to assure donor privacy, along with a re-identifiable process for specific conditions where clinically relevant information becomes known and can be provided to the patient; and (v) to include established governance structures and procedures (e.g., consent) that protect donors’ rights and stakeholder interests [5]. In parallel with improvements in sample management, data collection, and the increased use of biological samples for research purposes, it has become necessary to protect the patients and fulfill all the requirements of privacy, confidentiality, and human subject protection during sample sharing [6]. Consequently, modern biobanks function as complex infrastructures where clinicians, biologists, nurses, technicians, and bioethicists work together with the goal of guaranteeing the right to use human biological materials.

The aim of this manuscript is to provide a basic understanding of biobanking over time and describe how biobanks became essential structures for modern medical research. The first section provides a general overview on the evolution of biobanking, including the introduction of cell lines and specimen biobanks. The second shows how the collection, processing, and storage of human biological samples is evolving, highlighting the procedures performed in the workflow for different types of biological samples (e.g., tissues, cells, blood, DNA/RNA); this section also addresses the need to harmonize procedures related to biobanking. The final section describes the International Organization for Standardization (ISO) standards as international procedures to be followed to harmonize the data obtained from biological samples; this allows data comparison within of a vast network of biobanks. Below a dedicated section addresses associated bioethical aspects. We describe the history of bioethics in relation with the use of human biological samples, citing international documents that represent milestones for protecting the rights of each individual. Subsequently, we highlighted the main current bioethical issues in the field of biobanking, which continue to be a matter of debate. We also outlined the biobank international infrastructures dedicated to biobanking (BBMRI-ERIC) and then describe the collection, processing, storage, and sharing management of biological samples. Finally, we focus on recently established imaging biobanks. These are not merely a collection of bioimages associated with other patient clinical data; rather, they involve advanced computer technologies where image data, metadata, and raw data can be used for imaging measurements and biomarker extrapolation. These new biobanks can contribute to develop innovative research fields such as radiomics and radiogenomics. The first one extracts imaging features from bioimages (e.g., derived from modern computed tomography [CT] or magnetic resonance [MR] instruments) that can be used as prospective disease biomarkers. The second correlates imaging with genomic data, often obtained through high-performance molecular techniques such as next-generation sequencing (NGS) technologies, DNA sequencing, and microarrays. Both radiomics and radiogenomics aim to ameliorate patient management using a non-/or minimally invasive approach.

**Fig. 1:**
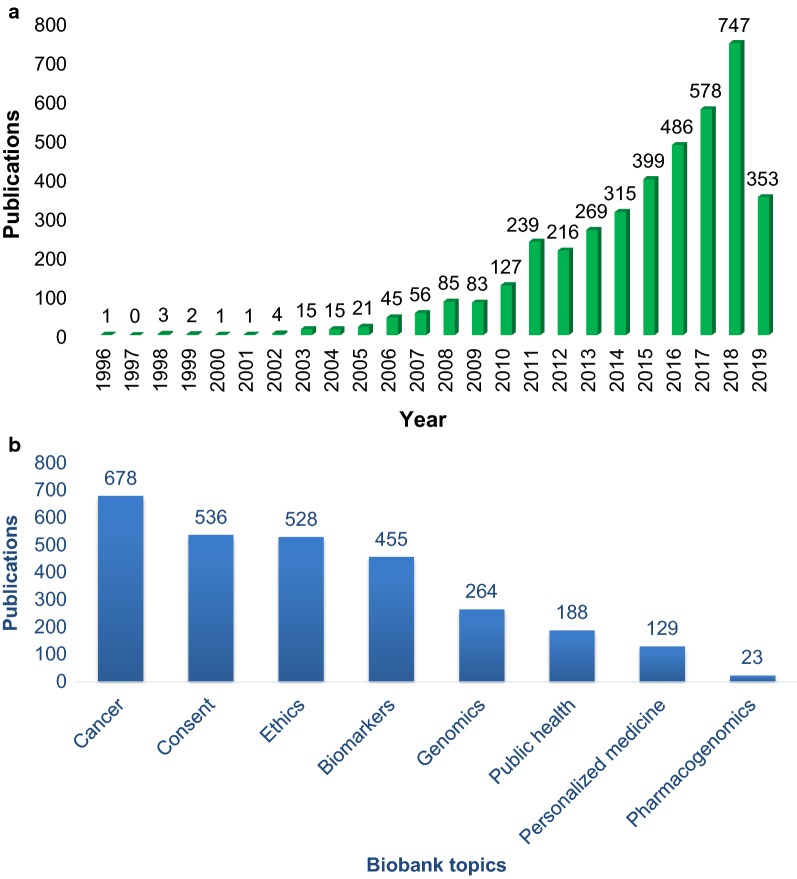
Graphical representation of the number of publications related to biobanking obtained from PubMed. **a** Shows the number of publications over time. **b** Shows the number of publications classified for: Cancer, Consent, Ethics, Biomarkers, Genomics, Public health, Personalized medicine and Pharmacogenomics (April 8, 2019)

A literature review was conducted on April 8, 2019 using the online database PubMed accessed through the Bibliosan platform of which the IRCCS SDN is a member (research project: “Design and operational implementation of the Library System of Italian Biomedical Research Institutes” promoted by the Ministry of Health, Italy, 2003). An increasing number of published scientific papers mentioned biobanks over the last 15 years (total = 4061 results, Fig. 1a). The initial screening was conducted based on the identification of specific keywords (Table 1). Our search criteria used the words “biobank” OR “biobanking” each combined with “cancer “AND “consent,” AND “ethics,” AND “genomics,” AND “public health,” AND “personalized medicine,” AND “pharmacogenomics,” AND “biomarkers” (Fig. 1b).

**Table 1:** Literature review results using the PubMed database

Keywords	Search mode	Results
Biobank or biobanking	((biobank[Text Word]) OR biobanking[Text Word])	4061
Biobank or biobanking and cancer	((biobank[Text Word]) OR biobanking[Text Word]) AND cancer[Text Word]	678
Biobank or biobanking and consent	((biobank[Text Word]) OR biobanking[Text Word]) AND consent[Text Word]	536
Biobank or biobanking and ethics	((biobank[Text Word]) OR biobanking[Text Word]) AND ethics[Text Word]	528
Biobank or biobanking and biomarkers	((biobank[Text Word]) OR biobanking[Text Word]) AND biomarkers[Text Word]	455
Biobank or biobanking and genomics	((biobank[Text Word]) OR biobanking[Text Word]) AND genomics[Text Word]	264
Biobank or biobanking and public health	((biobank[Text Word]) OR biobanking[Text Word]) AND public health[Text Word]	188
Biobank or biobanking and personalized medicine	((biobank[Text Word]) OR biobanking[Text Word]) AND personalized medicine[Text Word]	129
Biobank or biobanking and pharmacogenomics	((biobank[Text Word]) OR biobanking[Text Word]) AND pharmacogenomics[Text Word]	23

This table shows the search criteria (keywords and search mode) used for screening PubMed database. Results shows the number of scientific articles obtained

The goal of this review is to highlight four primary topics related to biobanking: (i) the evolution of biobanking, (ii) bioethical issues related to biobanking, (iii) biobank organization, and (iv) imaging biobanks.

## Main text

### The evolution of biobanking

The last several decades have seen tremendous improvements in the collection and storage of human samples, allowing the worldwide scientific community to obtain very important results in the field of medical research. Today we can collect, store, and preserve tissues, cells, DNA, proteins, and other subcellular components on a long-term basis [7]. In this section, we will discuss how biobanking evolved over time both for cell lines and specimens and how biosamples need to be qualified. We will also discuss the decentralized evolution of biobanking and the consequent requirement of standard procedures for the harmonization of sample collection and usage.

#### Cell line biobanking

The history of cell line biobanking started with generation of the HeLa cell line in 1951 at Johns Hopkins Hospital, where the medical staff obtained the first cancer cell line from a patient named Henrietta Lacks (HeLa). Cancer cells were obtained without her consent after surgery and cultured in the lab using an experimental approach that made the cells immortal, thereby achieving, for the first time, an in vitro cancer model system for research [8]. The HeLa cell line is used worldwide in the research laboratories because it is easy to grow and offers an optimal and stable model system for in vitro research experiments. Thanks to the epochal HeLa cell line, scientific results have been gained with crucial advantages for global health, including the development of polio vaccines [9]. The success of the HeLa cell line encouraged the stabilization and use of immortalized cell lines for medical research, particularly during the 70 s and 80 s when several continuous cancer cell lines were established. The growing number of newly generated cell lines underscored the need for an impartial organization that could guarantee the origin and quality of each model system. In this way, the evolution of cell line biobanks highlights the importance of standardizing technical procedures and ensuring data reproducibility in medical research. The first step for procedure harmonization was the institution of official cell line biobanks. The first was the American Type Culture Collection (ATCC) that was established with the aim of providing certified model systems to the scientific community. Currently, major cell line repositories include: (i) the ATCC (USA), (ii) the Leibniz-Institute DSMZ (Deutsche Sammlung von Mikroorganismen und Zellkulturen); (iii) the European Collection of Cell Cultures (ECACC); (iv) the Japanese Cancer Research Resources Bank (JCRB); RIKEN BioResource Center (Japan); and (v) the Korean Cell Line Bank (KCLB). These repositories still represent international references that guarantee authentic and highly controlled in vitro model systems for medical research, with appropriate certification including disease-associated data, karyotyping, immunoprofiles, unique molecular or genetic alterations, growing conditions, and mycoplasma testing [10]. Expanding the scientific interest toward other sources of biological material, it is important to consider the evolutionary process of biospecimen biobanks.

#### Specimen biobanking

The first specimen biobanks started as university-based repositories for specific research projects. They were established by researchers with access to patient populations who took advantage of the availability of “left over” aliquots to be stored for immediate or future use. Samples were stored in one or a few freezers, and associated data were recorded in a laboratory notebook or basic database [11]. However, albeit these small collections lack the big data (clinical, genomic etc.), standard operative procedures and automated sample processing, they could be seen as the initial phase of the evolutionary process of human biobanking.

Over the time, technological advances, notably automated sample processing, computerization, and the advent of the World Wide Web revolutionized the management of biobanks, which developed into more complex units. According to The Research Centre Institute for Prospective Technological Studies of the European Commission (EUR 24361 EN-2010), 40% of European biobanks started during the 90 s, while 37% began after 2000. This trend coincided with completion of the human genome sequencing project, which led to genome-wide association studies focused on identifying disease susceptibility genes and diagnostic biomarkers. After this event, biobanking institution grew exponentially, assuming a unique role in contemporary scientific research. Indeed, different research programs have benefited from biobank specimens. One famous case is the development of the trastuzumab antibody (Herceptin). Starting from the evaluation of tumor specimens stored at National Cancer Institute’s Cooperative Breast Cancer Tissue Resource, it became one of the drugs effectively used to treat specific subtypes of breast cancer [12]. More recently, biobanks played a critical role in the development of Cancer Genome Atlas (TCGA), a publicly funded project that aims to create a comprehensive “atlas” of cancer genomic profiles by cataloguing major cancer-causing genomic alterations [13]. Thanks to human specimens with associated clinical data, it is possible to analyze large cohorts of over 30 tumors with large-scale genome sequencing. This approach has led to the identification of several novel molecular alterations in cancer, and tumor subtypes can be classified according to distinct genomic alterations, allowing a precision medicine approach for patient care.

#### Biobank classification

Although the literature offers different classifications, a more general distinction can be made between population-based and disease-oriented biobanks. The first focuses on studying the possible future development of common and complex diseases, while the second is based on specific diseases, primarily cancer [4, 14, 15].

Population-based biobanks, such as Danish National Biobank [16], Estonia Biobank [17, 18], and UK Biobank [19], collect biological samples primarily from volunteer—without specific inclusion or exclusion criteria. The aim of population-based biobanks is to examine the role of individual genetic susceptibility and exposure to external factors in the development of specific diseases by combining molecular data with other associated data (clinical data, laboratory test results, questionnaire data, imaging data).

Disease-oriented biobanks were created to promote the study of human illness pathogenesis to identify possible therapeutic strategies. Thanks to the integration of different data encompassing a large number of biological samples, the research groups are able to develop large-scale research projects and collect information on the well-being of the examined population. In 1982 at the University of California, San Francisco (UCSF), an intervention strategy against AIDS was promoted with the establishment of the AIDS Specimen Bank. Researchers in epidemiology, infectious diseases, and pathology worked together to help discover the causative agent for AIDS with the goal of introducing new therapy protocols. The resulting network is now a major resource for investigators at UCSF and represents a global reference in this research field [20].

The advent of the World Wide Web considerably changed biobank administration, which opened new frontiers. By cataloguing extensive information about human samples, including annotation and location tagging, these virtual biobanks enable easy information sharing without the need to physically use biological samples, permitting simple sharing of medical data and allowing the development of networks for better cooperation between national and international biobanks [21]. Furthermore, this process negates the need to transport samples between two locations for a specific study, minimizing the risk of contamination.

#### Decentralized evolution of biobanking

While biobank foundation was an important medical research milestone, it is important to consider that their evolution has been decentralized. The different national directives established by local governance (data protection rules) [22] and, from a technical point of view, the different methods for collection/storage/qualification of basic data, have generated a high level of heterogeneity between biobanks [23]. These aspects could be potential barriers to the overall goal of an international research framework that aims to facilitate access to human biological materials. The life cycle stages of biosamples (intended as the collection, accession, acquisition, identification, preservation, long-term storage, quality control (QC), transport, and disposal of biomaterials) are a major source of heterogeneity. Result comparisons become difficult when different procedures for biosample management are applied. For this reason, harmonization of at least minimum standards for access and compensation need to be reached to facilitate large-scale, efficient use of human biological samples [24].

#### Qualification of biosamples

Qualification is the process used to verify the quality of biospecimens collected into a biobank. It is crucial for basic data and avoiding the introduction of uncontrollable variables that could make biospecimen samples incomparable in terms of quality. Considering that downstream analysis depends on the biobank research area (population-based or disease-oriented) and that different QC assays are used for biospecimen qualification, high heterogeneity between biobanks has developed over time.

There are two main approaches for qualification: the first includes the collection of biosamples with careful pre-analytical annotation (SPREC) [25], the second refers to retrospective collections with appropriate QC and sample qualification or quality stratification. The term “sample qualification” refers to the examination and validation of a single biospecimen or a collection on the basis of objective analytical parameters. “Quality stratification” refers to a process of examining and classifying biospecimens in different categories corresponding to specific in vivo parameters (e.g., protein content) or ex vivo pre-analytical conditions (e.g., pre-centrifugation conditions) [26].

Qualification methods and parameter measurements are strictly dependent on the downstream analysis they are collected for. Testing parameters of biosamples are specific for disease areas or for a specific downstream analytical platform, so not all parameters are tested everywhere, and parameter testing depends on the locally adopted standards. For example, a coagulation disease-oriented biobanking could test different parameters (e.g., fibrinogen, prothrombin, plasminogen activator inhibitor type-1) with different methods (clot detection, enzyme immunoassay [EIA], fluoro-immunoassay) for QC of plasma biosamples. Furthermore, different test methods entail different sensibility/specificity and threshold parameters, making data reproducibility difficult. Together with the different parameters that can be measured to qualify the same biosample typology, there are some measurement methods that are difficult to objectify, for example immunohistochemistry (IHC), immunocytochemistry (ICC), or microscopy for the qualification of tissue samples, cell suspensions, cell lines, or stem cells [27].

#### Storage/collection in biobanking

This paragraph aims at discussing some aspects related to the heterogeneity for collecting tissues, cells, blood, and nucleic acids.

##### Tissue/cells biobanking

Procurement of biospecimens for biobanking has long been an unstudied issue without standard practices for regulating pre-analytical steps. Human biological samples include a vast range of tissues, biospecimens, organs, body parts, extracted DNA or RNA, blood, bodily fluids, cell lines, cell suspensions, plasma, and so on. Today, a large number of research projects focus on genome, transcriptome, proteome and metabolome areas conducted on tissue samples of patients with a defined clinical and pathological diagnosis or on cell lines/suspensions derived from patients’ blood. Human tissues are usually obtained from surgeries and autopsies; immediately after surgery tissues undergo histopathological examination by pathologist. In this step, the most accepted clinical practice for preventing tissue degradation and diminishing unwanted enzymatic activity is fixing the tissue usually with neutral-buffered formalin. Before the advent of technologies that allow partial or complete evaluation of the genome, epigenome, transcriptome, metabolome, and proteome components, formalin-fixed paraffin embedded (FFPE) tissue was the specimen usually collected in biobanking databases, and frozen biospecimens were used in research programs. The “next generation” era has revealed several disadvantages in the use of FFPE tissue for molecular/genetics and protein studies. Fresh or frozen tissue tends to have much better-quality DNA and RNA than formalin-fixed tissue, therefore fresh or frozen tissue is the most appropriate sample for whole-genome amplification, whole-genome sequencing, and cDNA microarray analysis [28]. Considering the large amount of FFPE tissues biobanked over time in biobanking and that it is the most available tissue sample derived from clinical practice, some technologies have been modified to test FFPE samples at room temperature [29]. Furthermore, DNA and protein integrity are maintained in tissue blocks, while stored IHC slides have decreased antigenicity over time [30–33]. Nevertheless, biobanks will likely have to store increasing numbers of frozen biospecimens to avoid limitations in the intended research.

Tissue freezing is another preclinical step that could determine heterogeneity and decentralization of biobanking. Usually, surgically resected tissue stays at ambient room temperature before being stabilized. The amount of time that passes before stabilization is called “warm ischemia,” and it is a crucial variable that influences degradation before fixation. Furthermore, warm ischemia time is not always recorded since the attention is rightly focused on successful surgery. Some biobanks send a technician into operating room with a liquid nitrogen container to reduce the time between surgery and sample stabilization, but this practice is not widely used. Keeping the tissue at a cold temperature immediately after surgery could be the right way to ensure good specimen and, consequently, an additional step for standardization. Storing temperature conditions at the time of collection and during maintenance are also pre-analytical features that could affect basic data heterogeneity. In the past, all types of samples were kept at − 20 °C for both short and long-term storage. Today, the standard temperature for storage of tissues and cells are between − 80 °C and − 150 °C. Ultra-low temperatures preserve the integrity of proteins, DNA, RNA, and cellular components, even if the range of storage temperature does not guarantee the stability of every specimen type. A temperature of − 80 °C is now the standard for preserving human tissues/cells, even if some authors recommend liquid nitrogen, in particular the vapor phase stage (− 150 °C) over the liquid phase (− 196 °C) because of the risk of contamination by floating tissue fragments [29].

Freezing causes several adverse effects in living cells and tissues, so cryoprotectants are commonly used to prevent or reduce cryoinjury. Two major groups of cryoprotective agents are used to facilitate good recovery of viable and functional cells following cryopreservation. Dimethyl sulfoxide (DMSO), glycerol, and 1,2-propanediol, can penetrate the cell membrane [34], and large molecules like polysaccharides, carbohydrates, or glycoproteins that are not able to pass through the membrane [35, 36]. The use of DMSO is very common, and it has been one of the most efficient cryoprotectant; however, carbohydrates are often added to reduce its molar fraction and its cytotoxicity [37, 38]. Importantly, cryopreserving agent concentration should be optimized depending on the cell/tissue type to obtain the best survival rate after thawing [39]. This is especially crucial for bulk tissues where equal distribution of cryoprotective agent is not guaranteed because of the different heat and mass transfer effects during cryopreservation [35, 40]. Conventional cryopreservation media include fetal bovine serum, which contains a mixture of growth factors, cytokines, and other substances rendering its use forbidden in the establishment of a standardized cryopreservation protocol [41].

##### Blood biobanking

Blood is one of the most common biospecimens used in research. It is collected in tubes containing preservatives and additives depending on the specific downstream application and blood fraction needed (serum, plasma, white blood cells, red cells). While serum samples are generally obtained by collection in tubes containing a clot accelerator like thrombin or silica, plasma samples can be obtained using tubes containing several different anticoagulant additives. Most biochemical analyses are conducted on serum, while anticoagulated blood is used for DNA and RNA analyses. These practices can introduce heterogeneity among basic data. For example, citrate-stabilized blood yields higher DNA and RNA quality and more lymphocytes for culture compared to other anti-coagulants [42], while ethylenediaminetetraacetic acid (EDTA)-coated collection tubes would be preferred for protein assays and most of the analyses conducted on DNA molecules [43]. Heparin is appropriate for metabolomic studies but is not indicated for lymphocyte culture since it affects T cell proliferation [42].

Because of their lability, blood component stability is strictly dependent on the processing time. For example, protein integrity is guaranteed if plasma is immediately separated from blood [44, 45], while an optimal quality of extracted DNA from white blood cell blood samples can be processed after 24 h at 4 °C [46]. The optimal temperature for blood component storage varies depending on the specific analyte, marker, or molecule of interest. Generally, low (− 20 °C) and ultra-low temperature (− 80 °C) for short- and long-term storage, respectively, are the optimal conditions for maintaining the integrity and stability of every blood component [45, 47].

##### DNA/RNA

Molecular analysis is strictly dependent on the collection/extraction/storage modalities of DNA and RNA molecules. RNA is considered the most labile molecule, so a standard procedure for avoiding degradation is crucial. Different RNA yield and quality are achieved depending on the specimen type. Fresh frozen tissue is the ideal specimen for RNA extraction as genetic material is reduced in FFPE tissue due to cross-links between nucleic acids induced by formalin and the time interval between tissue resection and fixation [48, 49]. For good RNA quality, samples should be stored at − 80 °C without repeated freeze–thaw cycles.

DNA is more stable than RNA and can be kept at 4 °C for several weeks. A prolonged time between blood sampling and DNA extraction reduces DNA yield and integrity, so blood samples should be stored at − 80 °C if extraction cannot be performed immediately [50].

#### Procedure harmonization

The use of samples collected with standardized and validated protocols is a prerequisite to enable robust biological interpretation for data analysis and interpretation. For this purpose, the US National Cancer Human Biobank took a huge step forward in 2012 by introducing the first standard operating procedures (SOPs) for biobanks (http://biospecimens.cancer.gov/resources/SOPs). These were introduced according to the understanding that a lack of standardized, high-quality biosamples has slowed the progress of clinical research, and they still serve as an example for biobanks worldwide. The introduction of these specific procedures raised the need to harmonize national and international procedures concerning biobanking; this improvement is still one of the main goals of biobanks worldwide.

The publication of ISO 20387 (ISO 20387:2018 “Biobanking—General requirements for biobanking”) can be considered an important milestone for procedure harmonization at international level. ISO 20387 represents a reference standard for biobanks to adopt common strategies for the organization and processing of biological samples to achieve minimum standardization requirements. The general purpose of the ISO 20387:2018 guideline is to make available biological material capable of guaranteeing the reproducibility and comparability of scientific research results by standardizing the life cycle stages of the biological materials. Its point-by-point indications provide specific tools related to policies, processes, and procedures covering the life cycle of biological materials and their associated data from collection to storage, reception and distribution, transport and traceability, preparation and preservation of biological samples, QC of processes, and method validation and verification.

In conclusion, while critical advances in health care have been achieved using biospecimens from biobanks, more qualified biospecimens are still needed for discoveries to promote translational research. In this context, it is critical to standardize the processes of quality assessment, consenting, sample collection, storage, and access. The implication is clear: if more well-characterized, high-quality samples are available, research will advance and impact health care delivery.

### Bioethical issues related to biobanking

As mentioned earlier in the Henrietta Lacks case, the HeLa cell line is robust, immortal, easy to grow in culture, and was supplied to all scientists interested in studying this model system. In this way, HeLa cells proliferated around the world without consent or permission [51]. This infamous historical case has stimulated discussions in the scientific community on different themes, including a marked emphasis on informed consent (IC), commercialization, compensation, privacy and confidentiality, ethnicity, socio-economic status, health disparities, and familial implications of genetics information [52]. The introduction and development of sophisticated genetic technologies such as databases containing genotypic/phenotypic data and growing data sharing among national and international institutions have raised new questions regarding how best to inform and protect the participants of biobanking research. Bioethics for biobanking is an intense and growing field of interest; here, we present a brief overview of historical milestones in bioethics and discuss specific ethical issues related to biobanking including IC, ownership of biological material, and sustainability.

#### Historical milestones

Cases related to human rights violations such as those mentioned in the Nuremberg process (1945) on criminal medical scientists have raised the need for international regulations on human research. In 1964, the Declaration of Helsinki guidelines introduced the principles that “research protocols should be always reviewed by an independent committee prior to initiation” (an Internal Review Board or IRB) and that “research with humans should be based on results from laboratory animals and experimentation.” In addition, IC was declared a right for any clinical research participant. The Declaration of Helsinki represented a milestone in the preservation of human rights (last revision, 2013) and provides general guidelines for medical research on humans [53]. It sets ethical principles including the importance of protecting the dignity, autonomy, privacy, and confidentiality of research subjects, as well as obtaining IC for the use of identifiable human biological material and data. However, starting from the last revision of Declaration of Helsinki (2013), clinical research infrastructure networks have been evolving, and there are growing needs to manage large collections of human samples and related data and plan new research strategies and predictive analyses [54].

Although biobanks and health databases have enormous potential, they are considered dangerous to human rights due to the accessibility of sensitive data. To face this issue, the World Medical Association (WMA) published the Declaration of Taipei to provide guidelines on the collection, storage, and use of identifiable data and biological material beyond the individual care of patients [55]. This declaration defines a biobank as “a collection of biological materials and associated data” and a health database as “a system for collecting, organizing and storing health information.” It describes human biological materials as samples obtained from living or deceased individuals that can provide biological information about a specific subject analyzed. The collections in health databases and biobanks are both obtained from individuals and populations, giving rise to similar concerns regarding dignity, autonomy, privacy, confidentiality, and discrimination. This document suggests that there must be harmonization between advancing scientific progress and protecting individuals’ rights related to data obtained from their biological samples. It is the first international guideline to provide ethical directions about the complex issues that arise with activities associated with human databanks and biobanks [56].

#### Informed consent (IC)

Appropriate IC is one of the most intensely discussed topics within the context of biobank research [57]. The goal is to enable a competent individual to decide whether to participate in a research program. This means that it is crucial to understand all that the consent entails to ensuring that the individual’s decision is effectively “informed” [58]. Unfortunately, there is no international consensus due to differences among the legal system of each country. In Europe, the General Data Protection Regulation (GDPR-2018) provided important indications about IC. This legal framework sets guidelines for the collection and processing of personal information of individuals within the European Union (EU). It defines consent as “an unambiguous indication of a data subject’s wishes that signifies an agreement to the processing of personal data relating to him/her whereby that consent needs to be given in clearly defined.” Article 7 describes the essential conditions regarding consent (to be valid). Principal points are:

- Consent needs to be freely given;
- Consent needs to be specific, per purpose;
- Consent needs to be informed;
- Consent needs to be an unambiguous indication;
- Consent is an act: it needs to be given by a statement or by a clear act;
- Consent needs to be distinguishable from other matters;
- The request for consent needs to be in clear and plain language.

Despite the GDPR2018 guideline, optimization of consent for future studies is an argument of intense debate in the biobanking research field [59]. Indeed, classical IC that is focused on a specific research project is considered insufficient in biobanking. To date, most biobanks adopted a “broad consent” model [59], which is the agreement for utilization of his/her sample for current or future studies within a specified framework without the need for contacting the patient. Broad consent provides researchers sufficient flexibility to pursue a wide range of future, scientific agendas, but it implies that the patient acknowledges that he/she will not receive any feedback on incidental findings. With the development of information technology tools, a novel consent model has been proposed termed dynamic consent. This type of consent requires tools for easily accessible and constant contact with the patient to manage reconsent for each new project. Dynamic consent enhances autonomy and helps meet the desire for increased user participation in research programs. Indeed, dynamic consent uses modern communication strategies to inform, involve, offer choices, and obtain consent for every research project based on biobank resources. This approach focuses on new possibilities for constant communication and has the potential to increasingly involve participants in the use of their biological samples [60], but it also reveals several weaknesses such as therapeutic misconception [61]. Comparing dynamic and broad consent, the latter still represents a good ethical solution for biobank research. Nevertheless, improvements are needed in the broad model, and criticism can be met with adapting some of the modern communication strategies proposed for the dynamic consent approach [62]. More research in this field is required, especially to test and improve documents used in the IC process. According to Bossert et al. [58] it is important to develop bioethics research studies to: (i) assess IC readability; (ii) evaluate readers’ understanding and reactions; (iii) use test results to revise the IC document, and, consequently; (iv) re-evaluate the revised document. We believe that considering reader feedback and applying this proposed research scheme could be a valuable strategy for the development and improvement of IC and other kinds of written information.

#### Ownership of biological material

Research biobanks collect samples and data of human origin for their own research or that of third parties. Ownership of biological specimens is an important unsolved topic discussed in the literature. This argument presents ethical and legal issues in biobanking and, hopefully, all those involved in biobanking (patients/subject, researcher/clinicians, and institutional infrastructures) should be considered. Starting from the worldwide-recognized concept that no person can own another person, as this would constitute slavery and violate article 4 of the Universal Declaration of Human Rights [63], several biobanks have agreed to be custodians instead of owners of biological samples [64]. This statement is in line with that of International Agency for Research on Cancer (IARC) declaring, as a general rule, “no ownership of biological samples exists, and the biobank should assign ownership or custodianship based on national and institutional guidelines.” However, it is important to consider that local scientists who contribute to the biobank by collecting the biological samples could view the samples as “theirs.” Indeed, principal investigators may ask for priority access to specific collections or impose co-authorship in publication or veto rights to maintain a degree of control over biosamples due to scientific competition. This argument remains a theme of intense debate in the scientific literature and will be re-discussed in “Issues for sample access”.

#### Biobank sustainability

Biobank sustainability is another crucial aspect to be explored. A research project usually involves a study on a specific topic, which foresees predictable experimental costs, predetermined procedures, and a finite period of time. Conversely, biobanks have costs that are not fully definable and a financial management system that evolves over time. Biobanks are costly in regards to staffing, equipment, service contracts, consumables, and expertise [65]. To maintain sustainability, biobanks could run as business units. Indeed, some biobanks leverage the financial potential of their specimens and data, but this may lead to ethical and legal issues [66]. International biobanks have agreed that the human samples stored cannot be used for commercial purposes [67].

Most biobanks implement a cost recovery system by charging investigators access to specimens and related data. Several biobanks have developed self-financing strategies; for example, the UCSF AIDS Specimen Bank (ASB) developed a recharge methodology to recover costs associated with sample processing, storage, consumables, software and hardware maintenance, data management, and data sharing (https://cfar.ucsf.edu/cores/asb/services). Other examples of biobanks that use cost recovery strategies are: the Wales Cancer Bank (https://www.walescancerbank.com/cost-recovery-fees.htm), Australian Prostate Cancer BioResources (https://www.apcbioresource.org.au/for-researchers/apply-for-tissue-access/), and the Ontario Tumor Bank (https://ontariotumourbank.ca/researchers/access-fees).

Despite adopting this financial management system, the majority of biobanks are unable to fully recover their costs and have relied on the support of government policies and donations. A possible solution could be supporting biobanks mostly by local public bodies and partially by a cost recovery mechanism in order to favor efficient sustainability [68].

### Biobank organization

The main participants in biobanking are the individuals providing samples, communities, public bodies, and scientists [69]. The ultimate goal is to collect, store, and disseminate specimens and related data for medical research to hopefully improve health care for all. To this aim, biobanking facilities for collecting, processing, storing, and managing high-quality biological samples for medical research are increasingly endorsed worldwide. Notably, the processes involved from collection to storage and final use of samples should be carried out following standardized procedures that protect and respect patient confidentiality and rights. To this aim, international infrastructures play a key role in providing clear guidelines on IC procedures and ethical, legal, and social issues.

In this section, we will consider some critical aspects for the biobanking organization, including the role of infrastructures to coordinate biobank activity across countries; a general overview for sample collection, processing and storage; and procedures to access the samples.

#### Biobank infrastructures across countries

Research infrastructures are crucial for scientific research and are an important element of science policy. Biobanks play a central role in an increasingly complex research environment and are required to develop and implement accountable and transparent management procedures. Moreover, biobanking can be considered as an important transition from local research tools to complex international research infrastructures. Coordinating this process across countries is a critical process.

The coordination and structural components of biobanking infrastructures are diverse, with different goals, governance, and structures. Generally, biobanks are organized to acquire baseline and follow-up data of participants in cohorts classified by clinical features (e.g., pathology, disease stage, therapy, etc.), as well as sexual habits, work conditions, exposure to pollutants, and lifestyle habits. Moreover, data associated with samples are not limited to the moment of biospecimen collection but may be updated continuously by allowing its use in future studies. These considerations should motivate biobanks to have a dynamic organization. One of the primary needs in this field is the national and international standardization of procedures and management. In this context, The International Society for Biological and Environmental Repositories (ISBER) founded in 2000 (http://www.isber.org/) was one of the first and most important international organizations devoted to addressing the harmonization of scientific, technical, legal, and ethical issues relevant to repositories of biological and environmental specimens. ISBER was created by researchers, biobank managers (including those working with human, animal, plant, and environmental repositories and directors), National Institutes of Health representatives, bioinformatics managers, patient advocates, lawyers, and others interested in biobanking to share knowledge in the field. Indeed, ISBER fosters collaboration; creates education and training opportunities; provides an international showcase for state-of-the-art policies, processes, and research findings; and facilitates innovative technologies, products, and services. Each year, ISBER promotes new working groups to address areas such as biospecimen science, IC, informatics, rare diseases, and automated repositories. Moreover, they discuss important topics such as cost recovery, training, facilities, equipment, safety, quality assurance, QC, shipping, ethical issues, and specimen collection, processing, retrieval and culling [70].

After a preparatory phase project in 2008 [71], the European Biobanking and BioMolecular resources Research Infrastructure (BBMRI)-European Research Infrastructure Consortium (ERIC) was activated in 2011. The BBMRI-ERIC plays a critical role in health research and is composed of 19 European member states and 1 international organization, that is the IARC [71]. The BBMRI infrastructure was designed to operate across European countries. The goal is to link biobanks across Europe to foster cooperation and research that benefits both patients and European citizens. Indeed, it aims at improving interoperability of the existing population-based or clinically oriented biological samples from different European populations or patients with rare diseases. These collections include data on the health status, nutrition, lifestyle, and environmental exposure of study subjects. To fully realize the enormous potential of European biobanks for the benefit of society, the Stakeholders’ Forum was formed as a platform for clinical, ethical, and legal experts; the biotech and pharmaceutical industries; and patient advocacy groups. According to their suggestions, The BBMRI-ERIC seeks to develop standards and guidelines that properly respect individual values, such as protection of privacy and IC, with shared values of facilitated access to progress in healthcare and disease prevention [72]. According to Mayrhofer et al. the ultimate goal is that the BBMRI-ERIC will impact partnerships with patients/donors/participants, who know that their own tissues, samples, and personal data can yield discoveries and advances in medicine, diagnostics, and therapies [72]. In return, the BBMRI-ERIC is responsible for ensuring that the samples and data entrusted for research are used in the best way possible to advance knowledge and improve health care.

#### Sample collection, processing, and storage

**Fig. 2:**
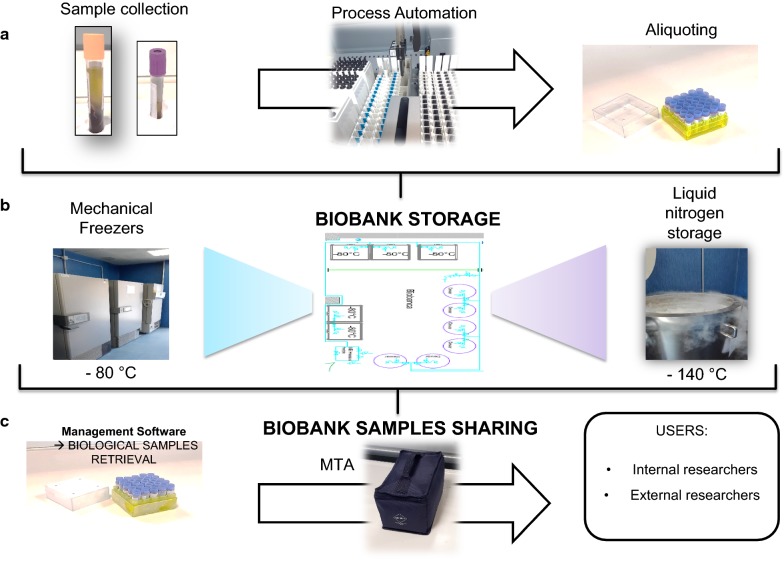
Hypothetical workflow model for collection, storage and distribution of samples in biobanking. **a** displays an example of automation of biological sample aliquoting. **b** shows a storage unit where biosamples can be stored in mechanical freezers or liquid nitrogen storage device. **c** displays the phases needed for samples sharing. A management software is needed for samples retrieval and an approved material transfer agreement (MTA) in case of both internal and external users before samples transferring

Biobanks are heterogeneous in their design (population or disease oriented) and use (epidemiology, translational, pharmaceutical research). They may contain data and samples from family studies, patients with a specific disease, clinical trials of new interventions, or they may be part of large-scale epidemiologic collections. Inevitably, data and samples are collected under various conditions and standards and for different purposes. Given the challenges of data collection and sample storage within studies, there has been little standardization across biobanks. A number of international initiatives to provide guidance and protocols were recently developed to address this issue [73]. The goal of procedure standardization and harmonization is to facilitate data sharing among different resources, thereby increasing effective sample size and statistical power, especially for rare diseases [73]. Quality programs need to be implemented to minimize the impact of variability on the integrity of the samples and, where possible, consideration should be given to future proofing the collection. In this context, in 2014, the ISO/Technical Committee (TC) 2761/Working Group (WG) 2 Biobanks and Bioresources was formed to ‘‘elaborate a package of International Standards in the Biobanking field, including human, animal, plant, and microorganism resources for research and development, but excluding therapeutic products.’’ The ISO/DIS 20387 was intended to be applicable to “all organizations performing biobanking activities, including biobanking of human, animal, plant and microorganism resources for research and development.” It provides a set of requirements to “enable biobanks to demonstrate that biobanking entities operate competently and are able to provide biological resources (biological material and associated data) of appropriate quality.” These requirements include personnel competence, method validation, and QC. It also includes general requirements for the competence, impartiality, and consistent operation of biobanks including QC to ensure that biological material and data collections are of appropriate quality. The ultimate aim of biobanking is to build and run quality-controlled storage facilities and infrastructures to enable future biomedical research. A proposed workflow model for the collection, storage, and distribution of biological specimens in biobanking is displayed in Fig. 2. Briefly, biological specimens are accepted and coded, and recorded on appropriate management software along with corresponding clinical information from patients. Successively, robotic process automation (Fig. 2a) can be used for sample aliquoting (preferably micro-aliquoting) before storage at low (− 80 °C) or ultralow (liquid nitrogen vapor phase, − 150 °C) temperature (Fig. 2b). The biobank software program should be able to manage biological samples and related information. To fulfill this purpose, it should have three basics features: (i) biological specimen management (consent management; non-conformity management; and biological resource history related to patients, samples, aliquots, derivatives, storage, and request management); (ii) traceability (follow-up for sample requests, annotation of collections with links to patient files [clinical, genetic, imaging data]); and (iii) interoperability (interface with temperature monitoring systems, interface with the hospital/laboratory to obtain further clinical data). Finally, the application of a material transfer agreement regulates biomaterials transfer between biobanks and recipients (research groups internal or external to the biobank infrastructure) to maintain specific quality and traceability standards for the samples [74] (Fig. 2c).

#### Issues for sample access

Human biological materials and related data stored in biobanks are valuable resources for biomedical research. Transparent, effective, and efficient governance structures and procedures for access, compensation, and priority setting are needed, but recent debates highlight challenges in their practical application [75]. Access to biological samples and related data have been much debated in recent years; indeed, access policies are neither standardized nor harmonized. Langhof et al. [76] recently highlighted the need for governance structures accepted by all stakeholders (patients/donors, researchers, research funders, public, and others) to ensure appropriate sample access for research. The authors proposed specific topics based on interviews with biobank experts on the BBMRI-ERIC infrastructure. Regarding biological sample sharing, they have raised different aspects that are debated but not yet harmonized, among the main ones:

- Access committee: An important element of sample access is the implementation of a scientific board to review access inquiries and project proposals. Access committees can be organized differently, basing their decisions only by majority vote or through a review system in which experts evaluate the scientific quality of research projects and consider the best use of the stored bioresources.
- Veto rights: as discussed in “Ownership of biological material”, local clinicians or principal investigators contributing to a particular collection (samples from patients affected by rare diseases or innovative clinical trials) may impose veto rights to maintain a degree of control over biosamples. Veto rights have been introduced by biobanks to deal with competition in the scientific community. However, the application of veto rights could be disputable from an ethical point of view because it implies a sort of ownership of biological material. To address this issue and allow equal access to the collection, some biobanks proposed a model with “shared ownership” [76]. It proposes that a biobank will provide free services to constitute the collection for a specific research project; however, half of the aliquots collected will be used by the biobank for additional research projects when necessary. Although this is a possible solution, the argument about sample ownership continues, especially in case of pharmaceutical companies, because the hope is that results obtained from human samples could yield profitable benefits [77].
- Prioritization of bioresources: it is important to consider that many human biological materials are finite resources. While DNA is often available in relatively large amounts for massive DNA analysis methods (e.g., NGS), postgenomic research (transcriptomics, metabolomics, and proteomics) mostly uses other biological materials such as plasma, serum, tissue, and urine. These biosamples might be valuable to a multitude of research projects and are normally rare. Related to the question of access decisions, another important issue is how to prioritize allocation of these relatively scarce biosamples.
- Compensation procedures for biobanks: another barrier to bioresource access is the recognition of biological sample sharing. The Bioresource Research Impact Factor (BRIF), could be a system for measuring the impact of each biobank in biomedical research and other forms of recognition such as including biobankers/researchers as coauthors of scientific papers [76, 78]. The idea behind the BRIF is to allow external researchers (e.g., researchers who are not affiliated with the biobank and/or have not contributed to the specific sample collection they are interested in) to access parts of the biosample collection; therefore, the biobank receives long-term recognition for the efforts to build-up and maintain its collection(s). To make this recognition visible, the BRIF requires a standardized citation of an individual (or several) biobank(s) in publications using its (or their) biosamples. However, the BRIF is still under development and has not been widely implemented; awareness of it is very limited even among biobank stakeholders in genetics. Moreover, the BRIF is focused solely on the biobank or its institutional host (e.g., a university hospital) and thus fails to acknowledge other actors who significantly contribute to biosample acquisition (e.g., clinicians and their contributing departments/institutes or individual researchers).

### Imaging biobanks

Biobanks effectively support health care allowing the discovery and validation of disease markers as well as novel therapeutic strategies [79]. A recent evolution of biobanking is the development of imaging biobanks [80]. In this section, we will discuss why bioimages from advanced computed tomography (CT), magnetic resonance (MR), and positron emission tomography (PET) [81, 82] need to be collected using a biobanking approach because they are useful for the identification and validation of a novel class of non-invasive biomarkers (i.e., imaging biomarkers [IB]).

#### Definition of imaging biobank

The growth of imaging biobanks is linked to the capability of high-throughput computing to extract numerous quantitative features from bioimages obtained with advanced CT, MR, and PET [81, 82] acquisition technologies. This promising area of research is defined as radiomics and focuses on evaluating extracted features as novel IBs for assessing physiological or pathological processes as well as pharmaceutical responses to a therapeutic intervention [83]. Some imaging features are already routinely used for patient monitoring in oncology, including tumor volume measurement, perfusion grade, Brownian motion of water molecules within a voxel of tissue, texture analysis, MR spectroscopy, stiffness, glucose metabolism, CT density, MR signal intensity, and MR fingerprinting. IBs could be very useful for patient management since they are non- or minimally invasive and could decrease the need for more invasive procedures like biopsy. Generally, IBs are considered as the expression of bio-signals because they are extracted from the analysis of an electromagnetic, photonic, or acoustic signal emitted by the patient. In this way, it is possible to consider IBs as a unique expression of a disease phenotype. According to Neri et al. imaging biobanks need to include data, metadata, raw data, measurements, and biomarkers derived from image analysis to allow feature extraction [80]. In addition, other disease-related factors (e.g., patient prognosis, pathological findings, genomic profiling, etc.) should be included for full validation and qualification in routine research or clinical use. This is important considering what was proposed in 2015 by the European Society of Radiology [84], which defined imaging biobanks as “organized databases of medical images, and associated IBs, shared among multiple researchers, and linked to other bio-repositories,” and suggested that “biobanks (which focus only on the collection of genotype data) should simultaneously come with a system to collect related clinical or phenotype data”. This definition highlights the roles that imaging biobanks play in ensuring standardization of data acquisition and analysis, accredited technical validation, and the transparent sharing of biological and clinical validation data. These steps are critical for effective translation of an IB into clinical practice according to Bonmatí et al. [85]. They highlight how clinical validation represents the bottleneck that selects useful and useless IBs. The awareness of imaging biobanks importance is gradually increasing, especially in the oncologic community, because they could be critical for crossing the translational gaps necessary to apply novel IBs in clinical practice [86].

#### Oncology oriented imaging biobanks

To date, most existing imaging biobanks have been oncologic oriented because IBs developed in diagnostic imaging are clinically used for oncologic purposes (e.g., glucose metabolism, tumor volume) [86]. An example of an oncological imaging biobank is the US-based Cancer Imaging Archive (TCIA), developed by the Frederick National Lab for Cancer Research [87]. TCIA is a service in which de-identified medical images from cancer patients are hosted in a large archive. The data are organized as “Collections” in which patients share a disease (e.g., lung cancer), an image modality (MRI, CT, among others), or a research focus. Digital Imaging and Communication in Medicine (DICOM) is the primary file format used by TCIA for image storage. Supporting data related to the images, such as patient outcomes, treatment details, genomics, pathology results, and expert analyses are also provided when available [87]. One major feature of TCIA is the open access; DICOM datasets and other “-omics” information are available for public download. Researchers can download datasets for many purposes, even to develop and test new IBs. There is currently no European counterpart to TCIA, but it would be desirable for imaging biobanks in Europe to become public to allow data sharing and stimulate the exploitation of imaging data from researchers. To this aim, it will be important to develop procedures to harmonize instruments for data collection and mining and perform comparative analyses.

#### Imaging biobanks for radiogenomics

**Fig. 3:**
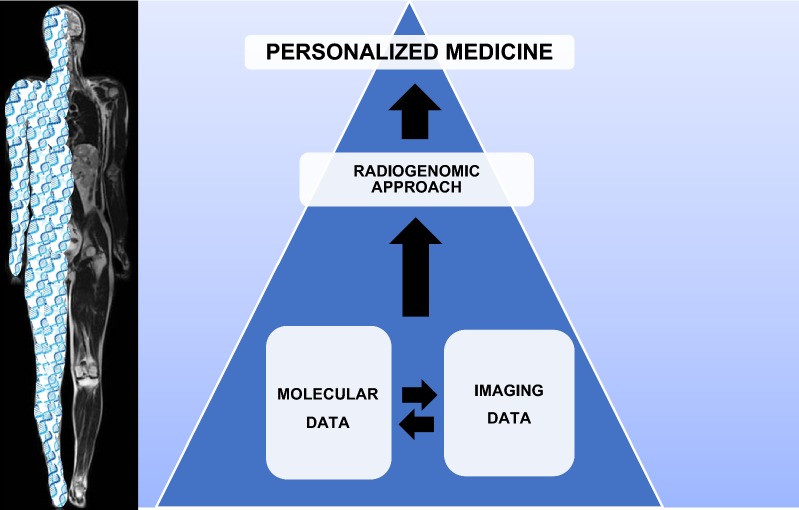
A schematic description of radiogenomic approach. The integration of molecular and imaging data is needed for a radiogenomic approach to the patient in a personalized medicine setting

The latest goal of multi-omics biobanks is integrating imaging and biological data to provide a deep association between phenotype and genotype with possible IBs. To obtain such integration, IB data from different “systems” should be in manageable formats (e.g., standardized quantitative MRI and PET protocols and measures, obtained and combined by separate or concurrent acquisitions) to be integrated with other “-omics” data, including the genomic profile of the same patient [88]. In this context, the bioinformatics approach plays a pivotal role in analyzing, correlating, and interpreting a large amount of data that cannot be managed with only on human capabilities. Based on these considerations, the immediate purposes of imaging biobanks are to enable generation of IBs for use in research studies and support biological validation of existing and newly identified IBs. Thanks to the development of imaging biobanks and the technological tools required for radiomic analyses, it is possible to integrate radiomic features with genetic data in a novel research approach that is defined as radiogenomics. This term applies in two different fields of study: “radiation genomics” and “imaging genomics.” Radiation genomics refers to the study of genetic variation (e.g., single-nucleotide polymorphisms) in relation to a cancer patient’s risk of developing toxicity following radiation therapy. It is also used in the context of studying the genomics of tumor response to radiation therapy. Imaging genomics refers to the extraction of IBs that can identify disease genomics, especially cancer without a biopsy sample [89]. Radiogenomics represents the evolution of radiology–pathology correlation from an anatomic–histologic level to a genetic level [90]. Imaging features are correlated with genomic data often obtained through high-performance molecular techniques such as NGS technologies, DNA sequencing, and microarray. Radiogenomics can better characterize tumor biology and capture intrinsic tumor heterogeneity with relevant implications for patient care. Existing radiogenomics studies were mainly concerned with oncologic diseases such as glioblastoma multiforme [91], lung cancer [92], prostate cancer [93], and breast cancer [94]. In this context, the increasing use of bioresources from certified biobanks associated with imaging will allow the merging of these two data types to improve patient management and provide personalized medicine as hypothetically conceived in Fig. 3. In this scenario, the use of hybrid biobanks that integrate imaging and molecular data is an interesting potential approach to improve clinical management on a personalized background.

## Conclusions

Biobanking represents a new and innovative research field that involves international infrastructures (e.g., BBMRI and ISBER) and government agencies in recognition of the need to adopt best practices and provide scientific, ethical, and legal guidelines for the industry and public health [26, 71]. Indeed, it is important to consider the growing demand for high-quality and clinically annotated biospecimens due to genomic, post-genomic, and personalized medicine research activities [3]. When facing these research challenges, it is important to consider the major problems related to the decentralized evolution of biobanking, such heterogeneous procedures for specimen collection and storage and ethical and legal issues related to specimen access [75, 79]. Biobank sustainability is another matter of debate since they need to be funded for decades, with rising costs for personnel, equipment, sample storage, and establishment of new (standard) methods [67]. A novel field is that of imaging biobanks [80]. In these units, diagnostic images generated with cutting-edge imaging technologies can be exploited by high-throughput computing to extract radiomic features [82]. These can be used as non- or minimally invasive biomarker; however, several translational gaps need to be fulfilled before their validation in clinical settings. To this aim, imaging and biological biobanks need to be linked to correlate patients’ clinical data and known biological biomarkers [90]. Furthermore, procedure harmonization is essential to guarantee the reproducibility of the extracted features and the application of IBs in multiple diagnostic contexts [85]. These steps are critical to assess the usefulness of IBs in a clinical setting. Imaging biobanks linked to biological samples and patients’ clinical information can be considered as a new frontier in biobanking. They could lead to the generation of multi-omics biobanks, where radiomic data could be integrated with genomics, proteomics, or metabolomics findings for an innovative and personalized approach to disease treatment [90]. In conclusion, we believe that the future of medical research is strictly related to that of biobanking, which will rely on the number and diversity of available biospecimens and bioimages, cost management and realization, patient and citizen participation, and national and international institution governance. An effective biobank should offer high-quality and affordable biospecimens for planning research programs that will benefit everyone.

## Data Availability

Not applicable.

## Abbreviations

ATCC: American Type Culture Collection
BBMRI: Biobanking and Biomolecular Resources Research Infrastructure
BRIF: Bioresource Research Impact Factor
CT: Computed Tomography
DICOM: Digital Imaging and Communication in Medicine
DMSO: Dimethyl Sulfoxide
DSMZ: Deutsche Sammlung von Mikroorganismen und Zellkulturen
ECACC: European Collection of Authenticated Cell Cultures
EDTA: Ethylene diamine tetra-acetic acid
EIA: Enzyme Immune Assay
ERIC: European Research Infrastructure Consortium
FFPE: Formalin-Fixed Paraffin Embedded
GDPR: General Data Protection Regulation
IARC: International Agency for Research on Cancer
IB: Imaging Biomarker
IC: Informed Consent
ICC: ImmunoCytoChemistry
IHC: ImmunoHistoChemistry
ISBER: International Society for Biological and Environmental Repositories
ISO: International Organization for Standardization
JCRB: Japanese Cancer Research Resources Bank
KCLB: Korean Cell Line Bank
MR: Magnetic Resonance
NGS: Next Generation Sequencing
PET: Positron Emission Tomography
QC: Quality Control
SOP: Standard Operating Procedures
SPREC: Standard PRE-analytical Code
TCGA: Cancer Genome Atlas
TCIA: The Cancer Imaging Archive
WMA: World Medical Association
